# Socio-economic risk factors for early childhood underweight in Bangladesh

**DOI:** 10.1186/s12992-018-0372-7

**Published:** 2018-05-30

**Authors:** Tuhinur Rahman Chowdhury, Sayan Chakrabarty, Muntaha Rakib, Sue Saltmarsh, Kendrick A. Davis

**Affiliations:** 1 Department of Economics, Shahjalal University of Science & Technology, Sylhet Kumargaon, Sylhet, 3114 Bangladesh; 2 Institute for Resilient Regions, University of Southern Queensland, QLD, Springfield, 4300 Australia; 3 School of Teacher Education and Early Childhood, University of Southern Queensland, Springfield Campus, Springfield, Australia; 4 School of Medicine, University of California, Riverside, USA

**Keywords:** Determinants, Underweight, Bangladesh, Child, Early childhood

## Abstract

**Background:**

Underweight is a major cause of global disease burden. It is associated with child mortality and morbidity, and its adverse impact on human performance and child survival is well recognized. Underweight is a major public health problem in Bangladesh, which is amongst the highest underweight prevalent countries in the world. The objectives of our study were to determine the national and regional prevalence rates of underweight and severe underweight in Bangladesh, and to investigate the association of socioeconomic and demographic factors with child underweight and severely underweight among children under the age of five living in Bangladesh.

**Methods:**

We performed a cross sectional study using Multiple Indicators Cluster Survey 2012–13, Bangladesh data on 17,133 children under 5 years of age. Weight-for-age Z scores based upon World Health Organization (WHO) guidelines were used to define child underweight and severe underweight. The association of underweight and severe underweight with household socioeconomic factors and demographic characteristics was investigated using binary logistic regression model.

**Results:**

An estimated 31.67% children were underweight and 8.81% children were severely underweight. Children of mothers with incomplete secondary education [Odds Ratio (OR) = 0.84, 95% CI: 0.75, 0.94] and mothers with completed secondary education [Odds Ratio (OR) = 0.77, 95% CI: 0.64, 0.93] were less likely to be underweight than children of uneducated mothers who had no formal schooling. A similar association exists for father’s education, children from households in the highest wealth index quintile had lower likelihood of underweight [Odds Ratio (OR) = 0.44, 95% CI: 0.37, 0.53] than children from households in the lowest quintile. Consumption of non-iodized salt had higher risk of severe underweight for children aged between 24 and 35 months [Odds Ratio (OR) = 2.32, 95% CI: 1.80, 3.00]. Other risk factors of child severe underweight included living in Sylhet division and increases in the number of children under the age of five in a household.

**Conclusion:**

Underweight was associated with lower parental education, household position in lower wealth index, living in Sylhet division and consumption of non-iodized salt. Strategies are discussed considering the relative importance of risk factors for child underweight.

## Background

Developing countries have the greatest prevalence of early childhood malnutrition despite recent rapid economic development [1–3]. Malnutrition is the prime culprit for early childhood death, causing the deaths of 13 million infants and children under the age of five every year [4]. There is a higher risk of dying among children who are mild to moderately malnourished compared with well-nourished children [5, 6]. The adverse impacts of malnutrition are not limited to school attendance rates and academic accomplishments, but also linked to decreases in the development of social skills among children [7, 8]. Malnutrition is associated with poorer academic performance in school-aged children [9] and lower survival capacity for a child in its adulthood [10].

Malnutrition status is investigated using three anthropometric parameters: height for age, weight for height and weight for age [11]. Height for age is used to identify stunting^1^ which represents linear growth failure for a child [12]. Weight for height is used to identify wasting^2^ which indicates an unusually body tissue and fat mass for a child relative to its height or length [13]. Weight for age is the WHO-recommended indicator to determine whether the child is underweight^3^ or not [11]. As underweight is a composite indicator that encompasses both stunting and wasting [14] implying that stunting, wasting or both can be reflected by underweight [15], our primary interest is to use weight for age indicator to measure early childhood underweight.

Underweight has become a major public health problem particularly in developing nations, and its destructive impact on human performance and child survival has been well recognized [16]. It is a prime concern as underweight is the leading cause of global disease burden [17]. Underweight is associated with increased risk of contagious disease such as diarrhea and pneumonia [18]. The World Health Organization (WHO) cites underweight as the single largest risk factor in developing countries to the burden of disease, [19] as an estimated 52.5% of all deaths among young children are attributable to underweight [18]. In the countries where child mortality is high, underweight is accountable for 15% of the total disability adjusted life years losses [17]. Underweight children are 8.4 times more likely to die and children who are suffering from moderate and mildly underweight are 4.6 times and 2.5 times respectively more likely to die before the age of five compared to children who are well-nourished [5].

In 2011, 101 million children globally were estimated as underweight indicating the prevalence rate of underweight as 16% [20]. The situation is worse in Bangladesh, where underweight prevalence rate was reported to be 41% [21]. There are many socioeconomic and biological factors that are associated with child underweight. Biological factors include lower birth weight [22], having an underweight mother or father, and having a mother or father of short stature. Among the socioeconomic factors, children with mothers who don’t have any formal education are at greater risk of being underweight [23].

Even though Bangladesh has one of the highest prevalence of underweight in the world, few studies have identified the socioeconomic factors responsible for child underweight [24–30]. Most of the studies conducted in Bangladesh suggested that maternal education is a strong and significant predictor for child underweight [24, 28, 29, 31, 32]. A recent study by Devkota et al. [28] established a positive association of WAZ (weight-for-age z score) with the level of maternal education. Mohsena et al. [24] explored the Bangladesh Demographic and Health Survey (2004) Data and found that higher levels of maternal education are associated with lower risk of underweight. In Bangladesh, paternal education was also found to be an independent determinant of child underweight by few studies [27, 33]. According to Das et al. [33], children of illiterate fathers had a 30% higher likelihood of being underweight than the children of fathers who had completed primary school. Another study that finds a positive association between paternal education and risk of child underweight [26].

Socioeconomic status like poverty is also a significant predictor of child underweight. Children coming from poor families are more likely to be underweight compared with children coming from wealthier families [4]. Despite the fact that Bangladesh has managed to achieve an enormous economic growth in the last 20 years along with massive improvements in educational achievement, still a large proportion of its population, nearly 43.3%, are living under the poverty line of 1.25 dollar per day [28]. Having such a large proportion of the population living in poverty, hence unable to afford nutritious food or access improved health care facilities, makes the task of reducing the country’s prevalence of underweight children extremely difficult. Indeed, a number of studies conducted in Bangladesh suggested household socioeconomic status as a significant determinant of child underweight, reporting that children from relatively higher socioeconomic condition had lower likelihood of being underweight [28, 33]. A study in Bangladesh carried out by Alom et al. [29] reported that children who came from the richest wealth index had 44% lower odds of being underweight compared with children from the poorest wealth index.

Among the socioeconomic determinants, most of the studies conducted in Bangladesh confirmed that mothers’ literacy status, choice of latrine and poor household income are the associated risk factors for underweight among children under five [25, 27, 29, 30]. Other risk factors estimated as significantly associated with the risk of child underweight are improper feeding practices, maternal antenatal and postnatal care, diarrhea of children within last 2 weeks [32]; living in rural area [30]; mothers having less exposure to media, children of mothers working outside home [31]; unimproved toilet facilities, and living in Sylhet division [33]. The risk of being underweight among under-five children was observed to increase with an increase in children’s age by several studies in Bangladesh. [29, 31, 33]. The causes for underweight are multidimensional and interrelated, and they often change from country to country, highlighting the importance of local research studies. Therefore, the objectives of our study is to determine the national and regional prevalence of underweight and severe underweight and their associated risk factors in Bangladesh.

## Method

### Data sources

The analysis was based on the Bangladesh Multiple Indicators Cluster Survey (MICS) 2012–13 data conducted by Bangladesh Bureau of Statistics along with UNICEF who provided technical and financial support. Data were collected from December 2012 to April 2013. The major objectives of the survey were to collect the most recent data on women and child health, household socioeconomic status, access to mass media, ICT improved sanitation and pure water, child nutrition, mortality and child development. The sample was selected using two-staged stratified cluster sampling approach. In the time period during which data were collected, there were seven divisions and 64 districts in Bangladesh and districts were defined as sampling strata [34]. The proportion of women with 4+ ANC visits, where the term ANC stands for antenatal care visit, was used as key indicator in estimating the size of sample. The sample size was determined using the following formula n= $\frac{\left[4(r)\left(1-r\right)(deff)\right]}{\left[{e}^2(pb)\left( Ave\  Size\right)(RR)\right]}$, where 4 = factor for achieving 95% confidence level; *r* = projected value of the indicator, which was assumed to be 26% and expressed as a proportion; *n* = sample size; deff = design effect for the indicator, and the value of deff was estimated to be 1.4 based on previous surveys; Ave Size = average household size, and it was assumed as 4.5; pb refers to the percentage of women in the total population, who gave birth in the last 2 years, and it was taken to be 4%; RR = anticipated response rate which was assumed to be 95% based on the previous survey experience [35]. In estimating r, maximum margin of error was allowed of ±8 to 9%, and thereby a sample size of 800 was decided for each district. For obtaining a more precise estimation result, 1000 samples were drawn from each of the 20 UNDAF districts. UNDAF (United Nation Development Assistance Framework) districts are those 20 districts that have been declared as prioritized districts by Bangladesh government. Twenty households per cluster were selected for the survey, taking in consideration various factors such as time, budget, design effect etc. Twenty households per cluster suggest that for drawing 1000 households as sample, 50 clusters would be needed in each of the 20 UNDAF districts, and 40 cluster would be needed for drawing 800 households as sample size in each of the remaining 44 non UNDAF districts. In this way, the resulting sample size would be 55,200. But 4 clusters were not visited due to unfavorable weather and, therefore, data was collected from 55,120 households. Clusters were selected using 2011 census frame. The first stage of sampling involved determining census enumeration areas required within each district, which were defined as primary sampling units. From 30 November, 2012 to 6 December, 2012, field operations were carried out in each enumeration area to make new listings of households; primarily because the 2011 census frame was not up-to-date. Experienced staff of the Bangladesh Bureau of Statistics (BBS) prepared the list of households in the field, and at the BBS headquarter, the listed households were numbered sequentially from 1 to n within each enumeration area. Here, Random Systematic Sample Selection Procedure was used to select 20 households in each enumeration area. Households included 23,402 children below 5 years of age and information on 20,903 under five children was collected, indicating a response rate of 89.3%. For the purpose of data collection, structured questionnaires were used. The questionnaires had four sections comprised of questions pertaining to household, women (age 15–49), children under five and water quality testing. Thirty-two data collection teams collected data, with each team having seven members including a supervisor and interviewers. The teams were provided with training for 14 days prior to collecting data. Recommended anthropometric tools were used to measure child weight and height [34]. Collected data from the field were entered using CSPro software and all questionnaires were entered twice to ensure the quality of data [35].

### Statistical analysis

There were 20,903 children under age 5 years about whom information was collected. However, 982 children were not included as there was no information for weight-for-age-z score. For the households with more than one child under the age of five, the youngest one was selected as the index child and the other children in the household were not considered for the study. Finally, data pertaining to 17,133 children below the age of five were subject to analysis. Weight-for-age-z score of WHO growth standard was used to create the underweight variable. Children were defined to be underweight if their weight-for-age-z scores were below minus two and severely underweight if their weight-for-age-z scores were below minus three. Data analysis was performed using Stata version 12.1. Child, paternal and household socioeconomic factors were included in the analysis as predictors of child underweight. Factors included child’s age, gender, geographic area, education level of mother, education level of father, regional division, wealth index quintile used as the proxy indicator of household socioeconomic status, number of household members, toilet facility, salt iodization test, whether the child drank plain water yesterday (yes/no), whether the child drank milk yesterday (yes/no), whether the child took vitamins yesterday (yes/no) and number of children in a household below the age of five. Wealth index score was computed by principal component analysis based on household assets and materials used to build house [35]. All households were then categorized into five categories depending on wealth score such as poorest, second, middle, fourth and richest. Toilet facility variable was created with four categories as flush latrine, pit latrine, hanging latrine and others. Descriptive statistics were created for all predictors as well as for response variable. As our dependent variable was dichotomous, underweight versus not underweight and severely underweight versus not severely underweight, predictors for the risk of child underweight and severely underweight were investigated using binary logistic regression model. Statistical significance was set at *p* < 0.05 and adjusted odds ratio was reported along with 95% confidence interval.

## Results

### Socioeconomic and demographic characteristics of the study population

The final analysis of 17,133 children estimated that 31.67% of these children were underweight including 8.81% as severely underweight. Table 1 demonstrated socioeconomic and demographic characteristics of the study population.

**Table 1:** Socioeconomic and demographic characteristics of the study population

	Total sample
	(*n* = 17133)
Characteristics	Proportion (%)
Household member	
<=3	14.29
4–6	63.64
> = 7	22.06
Age (months)	
0–5	10.93
6–11	10.80
12–23	21.88
24–35	20.60
36–47	18.75
48–59	17.04
Gender	
Male	51.67
Female	48.33
Area	
Urban	15.87
Rural	84.13
Division	
Dhaka	25.36
Barisal	9.64
Chittagong	19.53
Khulna	13.96
Rajshahi	10.28
Rangpur	13.01
Sylhet	8.21
Education of mother	
None	24.10
Primary incomplete	14.55
Primary complete	15.39
Secondary incomplete	34.59
Secondary complete or higher	11.37
Education of father	
None	34.79
Primary incomplete	15.73
Primary complete	13.75
Secondary incomplete	20.35
Secondary complete or higher	15.38
Wealth index quintile	
Poorest	29.06
Second	21.86
Middle	18.69
Fourth	16.52
Richest	13.87
Child drank plain water yesterday	
Yes	85.46
No	14.54
Child drank milk yesterday	
Yes	23.44
No	76.56
Child ate vitamins yesterday	
Yes	9.06
No	90.94
Toilet facility	
Flush latrine	23.94
Pit latrine	62.34
Hanging latrine	7.78
Others	5.93
Salt idolization test	
Not iodized 0 PPM	27.15
More than 0 PPM and less than 15 PPM	20.49
15 PPM or more	51.43
No salt in the household	0.92
Child underweight status	
Underweight	31.67
Severely underweight	8.81

The male to female ratio of children was 1069:1000. More than four-fifths of children came from rural area and around two-thirds of the surveyed children belonged to the households with four to six family members. More than one-fifth of the children were between the ages of 12 and 23 months, and approximately 11% of children were aged less than 5 months. The highest (25.36%) proportion of children lived in Dhaka division with the lowest from Sylhet division (8.21%). More than one-third of fathers did not go to school and likewise one-fourth of mothers did not have any formal education. Only 11.37% of mothers and 15.38% of fathers completed secondary education. Nearly, one third of the study children belonged to households of the poorest wealth quintile (29.06%) and only 13.87% children belonged to richest wealth quintile. On the previous day, approximately 86% children drank plain water 24% drank milk and 9.06% ate or drank vitamins. More than half of the households have iodized salt (51.43%). The majority of households had access to pit latrine (62.34%) and almost one-third of households had access to flush latrines.

### Risk factors for underweight

Table 2 lists the socioeconomic and demographic factors along with their association and the magnitude of impact on the risk for underweight among children below 5 years old using binary logistic regression analysis.

**Table 2:** Risk factors for total underweight in children aged less than 5 years

Factors		Underweight (%)	N	OR	95% CI
Household member	<=3	33.24	2449	ref.	
	4–6	31.54	10,904	0.91	(0.82–1.00)
	> = 7	31.03	3780	1.00	(0.88–1.13)
Age (Months)	0–5	21.10	1872	ref.	
	6–11	21.88	1851	1.17	(0.98–1.39)
	12–23	31.05	3749	1.98	(1.69–2.32)^***^
	24–35	36.91	3530	2.58	(2.20–3.03)^***^
	36–47	35.62	3212	2.44	(2.07–2.88)^***^
	48–59	34.77	2919	2.35	(1.98–2.77)^***^
Gender	Male	32.15	8853	ref.	
	Female	31.16	8280	0.96	(0.90–1.03)
Area	Urban	26.52	2719	ref.	
	Rural	32.64	14,414	0.96	(0.86–1.07)
Division	Dhaka	30.29	4345	ref.	
	Barisal	33.96	1652	1.04	(0.91–1.19)
	Chittagong	33.59	3346	1.11	(0.99–1.24)
	Khulna	26.55	2392	0.85	(0.75–0.96)^**^
	Rajshahi	30.14	1762	0.90	(0.79–1.02)
	Rangpur	32.17	2229	0.98	(0.87–1.11)
	Sylhet	38.52	1407	1.38	(1.20–1.60)^***^
Education of mother	None	39.55	4129	ref.	
	Primary incomplete	37.18	2493	1.02	(0.91–1.14)
	Primary complete	34.36	2637	0.96	(0.85–1.07)
	Secondary incomplete	27.19	5926	0.84	(0.75–0.94)^**^
	Secondary complete or higher	17.92	1948	0.77	(0.64–0.93)^**^
Education of father	None	38.25	5428	ref.	
	Primary incomplete	35.66	2454	0.98	(0.88–1.09)
	Primary complete	33.15	2145	0.97	(0.87–1.09)
	Secondary incomplete	27.81	3175	0.88	(0.79–0.98)^*^
	Secondary complete or higher	19.30	2399	0.72	(0.62–0.84)^***^
Wealth index quintile	Poorest	40.11	4979	ref.	
	Second	35.54	3745	0.90	(0.82–0.99)^*^
	Middle	30.78	3203	0.82	(0.73–0.91)^***^
	Fourth	25.48	2830	0.67	(0.59–0.77)^***^
	Richest	16.46	2376	0.44	(0.37–0.53)^***^
Child drank plain water yesterday	Yes	32.11	14,619	ref.	
	No	29.03	2487	1.27	(1.12–1.43)^***^
Child drank milk yesterday	Yes	26.94	4013	ref.	
	No	33.10	13,105	1.07	(0.98–1.17)
Child ate vitamins yesterday	Yes	28.69	1551	ref.	
	No	31.97	15,559	0.96	(0.84–1.09)
Toilet facility	Flush latrine	24.46	4100	ref.	
	Pit latrine	32.53	10,677	1.04	(0.94–1.16)
	Hanging latrine	41.86	1333	1.11	(0.95–1.30)
	Others	38.29	1016	1.02	(0.86–1.22)
Salt iodization test	Not iodized 0 PPM	36.54	4619	ref.	
	More than 0 PPM and less than 15 PPM	34.77	3486	0.96	(0.87–1.06)
	15 PPM or more	27.90	8750	0.90	(0.82–0.98)^*^
	No salt in the household	29.30	157	0.80	(0.55–1.16)
Number of under five children				1.02	(0.93–1.11)

^***^ *p* < 0.001; ^**^ *p* < 0.01; ^*^ *p* < 0.05

The analysis determined the following factors to be associated with the risk of underweight: age of children, division, education level of mother and education level of father, wealth index, whether the child drank plain water yesterday and finally the use of iodized salt by households. Children living in Khulna division were 15% less likely to be underweight [Odds Ratio (OR) = 0.85, 95% CI: 0.75, 0.96] compared with the children living in Dhaka division. Children from Sylhet division were more prone to the risk of underweight than any other division in Bangladesh with higher odds on underweight [Odds Ratio (OR) = 1.38, 95% CI: 1.20, 1.60] than the children from Dhaka division. Our results suggested child’s age as one of the major determinants of underweight. Children aged 12–23 months [Odds Ratio (OR) = 1.98, 95% CI: 1.69, 2.32] had around two-fold odds on underweight compared with children below 5 months of age. The risk for underweight was highest among the children who were in their early middle childhood. Children aged 24–35 months [Odds Ratio (OR) = 2.58, 95% CI: 2.20, 3.03] had over two-fold risk on underweight relative to the children less than 5 months of age. Children between the ages of 36–47 months [Odds Ratio (OR) = 2.44, 95% CI: 2.07, 2.88] and children between the ages of 48–59 months [Odds Ratio (OR) = 2.35, 95% CI: 1.98, 2.77] were more likely to be underweight compared with the children who were below 5 months of age. Children of secondary incomplete mothers had 16% smaller odds on underweight [Odds Ratio (OR) = 0.84, 95% CI: 0.75, 0.94] than the children of mothers with no formal education. There were 23% lower odds on underweight among children whose mothers completed secondary education [Odds Ratio (OR) = 0.77, 95% CI; 0.64, 0.93] than the children whose mothers had not attended school at all. As with mother’s education, an increase in the level of father’s education reduced the odds on child underweight. Children with father’s education secondary incomplete and secondary complete had 12% [Odds Ratio (OR) = 0.88, 95% CI: 0.79, 0.98] and 28% [Odds Ratio (OR) = 0.72, 95% CI: 0.62, 0.84] lower odds on underweight respectively in comparison with children whose fathers had no formal education. Children who didn’t drink plain water yesterday were 1.27 times more likely to be underweight [Odds Ratio (OR) = 1.27, 955 CI: 1.12, 1.43] compared with children who drank water the previous day.

Wealth index was also related to the risk of underweight. Children belonging to households which were in the middle category [Odds Ratio (OR) = 0.82, 95% CI: 0.73, 0.91] and in fourth category [Odds Ratio (OR) = 0.67, 95% CI: 0.59, 0.77] were less likely to be underweight compared with the children who belonged to the poorest households in the wealth index. There were 56% lower odds on underweight [Odds Ratio (OR) = 0.44, 95% CI: 0.37, 0.53] among the children who came from richest households than the children who came from poorest households. Children living in households where salt was found as adequately iodized (15 ppm or more) had a lower likelihood of underweight [Odds Ratio (OR) = 0.90, 95% CI: 0.82, 0.98] compared with children from those households where salt was found to be not iodized in salt iodization tests. Our study did not establish a relationship between child gender and underweight, nor did it establish that children from rural areas were more likely to be underweight compared with children from urban areas.

### Risk factors for severe underweight

Table 3 lists the results of the binary logistic regression analysis on the risk for child severe underweight associated with different socioeconomic and demographic factors.

**Table 3:** Risk factors for severe underweight in children aged less than 5 years

Factors		Severe Underweight (%)	N	OR	95% CI
Household member	<=3	8.82	2449		
	4--6	8.69	10,904	0.89	(0.75–1.05)
	> = 7	9.13	3780	0.98	(0.80–1.20)
Age (Months)	0--5	6.57	1872		
	6–11	6.86	1851	1.25	(0.94–1.66)
	12–23	9.02	3749	1.81	(1.40–2.34)^***^
	24–35	11.16	3530	2.32	(1.80–3.00)^***^
	36–47	9.74	3212	2.04	(1.56–2.65)^***^
	48–59	7.33	2919	1.48	(1.12–1.95)^***^
Gender	Male	9.25	8853		
	Female	8.33	8280	0.90	(0.80–1.00)
Area	Urban	6.91	2719		
	Rural	9.16	14,414	0.96	(0.80–1.15)
Division	Dhaka	8.63	4345		
	Barisal	9.87	1652	1.06	(0.86–1.32)
	Chittagong	10.10	3346	1.06	(0.89–1.26)
	Khulna	5.60	2392	0.68	(0.55–0.85)^**^
	Rajshahi	7.72	1762	0.86	(0.69–1.06)
	Rangpur	8.03	2229	0.82	(0.67–1.00)
	Sylhet	13.08	1407	1.34	(1.09–1.66)^**^
Education of mother	None	12.79	4129		
	Primary incomplete	10.87	2493	0.93	(0.78–1.10)
	Primary complete	8.99	2637	0.79	(0.66–0.95)^*^
	Secondary incomplete	6.77	5926	0.78	(0.65–0.92)^**^
	Secondary complete or higher	3.70	1948	0.69	(0.49–0.97)^*^
Education of father	None	11.81	5428		
	Primary incomplete	9.98	2454	0.93	(0.79–1.09)
	Primary complete	9.60	2145	1.02	(0.86–1.22)
	Secondary incomplete	6.46	3175	0.79	(0.65–0.95)^*^
	Secondary complete or higher	3.96	2399	0.66	(0.50–0.88)^**^
Wealth index quintile	Poorest	12.59	4979		
	Second	10.28	3745	0.93	(0.80–1.08)
	Middle	8.30	3203	0.80	(0.67–0.96)^*^
	Fourth	5.55	2830	0.51	(0.40–0.64)^***^
	Richest	3.11	2376	0.32	(0.23–0.46)^***^
Child drank plain water yesterday	Yes	8.65	14,619		
	No	9.77	2487	1.45	(1.21–1.73)^***^
Child drank milk yesterday	Yes	7.28	4013		
	No	9.28	13,105	0.94	(0.81–1.09)
Child ate vitamin yesterday	Yes	7.67	1551		
	No	8.93	15,559	1.01	(0.81–1.25)
Toilet facility	Flush latrine	6.44	4100		
	Pit latrine	8.73	10,677	0.91	(0.76–1.08)
	Hanging latrine	14.40	1333	1.01	(0.80–1.28)
	Others	11.81	1016	0.96	(0.73–1.26)
Salt iodization test	Not iodized 0 PPM	10.46	4619		
	More than 0 PPM and less than 15 PPM	10.33	3486	1.04	(0.89–1.21)
	15 PPM or more	7.38	8750	0.94	(0.82–1.08)
	No salt in the household	7.01	157	0.79	(0.42–1.47)
Number of under five children				1.17	(1.02–1.33)^*^

^***^ *p* < 0.001; ^**^ *p* < 0.01, ^*^ *p* < 0.0

Age of children was associated with the risk for child severe underweight with children aged 12–23 months having almost twice the odds on severe underweight [Odds Ratio (OR) = 1.81, 95% CI: 1.40, 2.34] than the children aged less than 5 months. Children aged 24–25 months had the highest risk for being severely underweight. Compared with children aged below 5 months, children aged 24–35 months [Odds Ratio (OR) = 2.32, 95% CI: 1.80, 3.00] had a more than two-fold risk of severe underweight. Furthermore, children between the ages of 48–59 months had also larger odds of severe underweight [Odds Ratio (OR) = 1.48, 95% CI: 1.12, 1.95] compared with their under-five counterparts. Children from the poorest households were at higher risk for being severely underweight. Children from households belonging to the middle category in wealth index had 20% lower likelihood of underweight [Odds Ratio (OR) = 0.80, 95% CI: 0.67, 0.96] and children from households that belonged to fourth category in the wealth index had 49% lower odds of underweight [Odds Ratio (OR) = 0.51, 95% CI: 0.40, 0.64] compared with the children from the poorest households. In comparison with the children from the poorest households, children from the wealthiest households had 68% lower odds of severe underweight [Odds Ratio (OR) = 0.32, 95% CI: 0.23, 0.46]. There were 32% lower odds on severe underweight among children living in the Khulna division [Odds Ratio (OR) = 0.68, 95% CI: 0.55, 0.85] and 34% higher odds on severe underweight among children living in the Sylhet division [Odds Ratio (OR) = 1.34, 95% CI: 1.09, 1.66] compared with children living in the Dhaka division. Improved mother’s education and father’s education levels were shown to be important protective factors against child severe underweight. Children with mothers’ primary education complete [Odds Ratio (OR) = 0.79, 95% CI: 0.66, 0.95] and secondary incomplete [Odds Ratio (OR) = 0.78, 95% CI: 0.65, 0.92] were less likely to be severely underweight compared with children whose mothers had no formal education. Children of mothers who had completed secondary education had 31% lower odds [Odds Ratio (OR) = 0.69, 95% CI: 0.49, 0.97] of being severely underweight compared with children whose mothers had not completed secondary education. Children had 21% lower odds of being severely underweight when their fathers had enrolled in secondary education compare to children whose fathers had no formal education [Odds Ratio (OR) = 0.79, 95% CI: 0.65, 0.95]. There were 34% lower odds of severely underweight among children with fathers who had completed secondary education [Odds Ratio (OR) = 0.66, 95% CI: 0.50, 0.88] compared with children whose fathers had no formal education. An increase in the number of children under the age of five in households by 1 was associated with 17% higher odds on severe underweight [Odds Ratio (OR) = 1.17, 95% CI: 1.02, 1.33].

## Discussion

Our study estimated 31.67% children below 5 years of age as underweight, with 8.8% children as severely underweight in 2011–2012 in Bangladesh. The present study findings indicated a concerning nutritional condition among children aged under 5 years, especially those aged 2–3 years. Child underweight and severely underweight were predicted by child’s age, regional division, education level of mother, education level of father, household position in wealth index, whether child drank plain water yesterday, and the presence of iodized salt in households.

Our study reveals maternal education as an important determinant of child underweight. According to our study findings, higher levels of maternal education are associated with lower odds of child underweight, which was supported by previous studies [24, 36, 37]. Likewise, maternal education was estimated to be an independent predictor of children being severely underweight. Children of secondary completed mothers had 31% lower odds of being severely underweight compared with children of uneducated mothers. Similar findings have been observed in earlier studies carried out in Bangladesh, which have shown that children of uneducated mothers were significantly more likely to be severely underweight than children whose mothers had a relatively higher level of education [25, 38–43]. There are some plausible explanations as to why higher maternal education reduces the chance of underweight and severely underweight among children under the age of five. First, education improves information processing capacity by the mother [44, 45]. For instance, an educated mother can read, interpret and apply health tips from newspapers and other sources, and is more likely to understand medical information such as medication levels suggested by doctor. These skills enable mothers to make informed decisions about their children’s nutrition and health care, leading to improved child health.

Second, maternal education is associated with an increase in the frequency of mothers’ antenatal care visits and their utilization of child health services, leading to decreased likelihood of underweight and severely underweight children. It has been evident from studies that the more educated mothers are the more likely they are to access child health facilities more regularly compared to less educated mothers [46–48]. Antenatal care is often associated with improved child nutrition [26], and a study that employed data from 17 countries, aiming at determining the relationship between maternal education and child survival, found that uneducated women represented 55% more non-use of antenatal services than their educated counterparts [49].

Third, there is general recognition that more educated mothers are more likely to earn more money [44, 50]. This implies increased capability to invest in their children’s health, and more possibilities for living in an environment with relatively higher food security. Finally, maternal education improves child nutritional status through its impact on appropriate feeding practices by mothers. Appropriate feeding practices have been documented as very crucial for improving nutrition conditions of children; particularly during their infancy [51], and a study conducted in Bangladesh revealed the association between improved feeding practices with education level of mother by demonstrating their findings that; compared with uneducated mothers, educated mothers were more concerned to breastfeed their children in protected place along with cleanness; that they were more aware to take breaks from the work at hand while breastfeeding their children and were more conscious ensuring hygiene in the time children were provided food [52]. Furthermore, higher level of education, completed by mothers, is associated with behavioral changes that improve child health. Women who have completed secondary education are more likely to have more knowledge of improved child care practices [53], are better able to take responsibility for their children during illness [54], and more likely to have greater control over household decision-making [55]. Even though we do not know exactly why increased maternal education is associated with lower likelihood of child underweight, the aforementioned result underscore the need for female education to reduce underweight prevalence among children.

Similar to maternal education, paternal education was a significant predictor of underweight and severe underweight. The result was supported by other studies that suggested low paternal education was associated with increased odds of child underweight [26, 27, 56, 57]. Education level of fathers is generally associated with household income due to the fact that more educated fathers are more likely to earn more money; indeed, father’s education is typically used as a proxy indicator of household income [58]. Data on Indonesian children suggested that preventive factors against child malnutrition, such as use of improved latrine, vitamin A capsules for children, access to health facilities from local health community clinics, and consumption of iodized salt, were strongly related with higher level of paternal education [12]. There is strong evidence that more educated men are more likely to marry more educated women. As a result, children of more educated fathers have an increased chance of having mothers who are also more educated. The view is supported by Grossman’s model of the demand for good health which claims that more efficient investment in health is associated with more education [59].

Another factor estimated to be significantly associated with child under-nutrition is the age of the children. Children below 5 months of age had the lowest risk of being underweight. One possible explanation could be that during this period children are still being breastfed, thereby gaining essential nutrition that renders them less vulnerable to malnutrition. On the other hand, study results indicate that children’s risk of being underweight increased with age, this risk reaching its peak between the ages of 24–35 months. This suggests that during that period of children’s development, they had more than double the risk for underweight than the children who were less than 5 months of age. The similar result was reported by a study which found the risk of being underweight and severely underweight as highest between the ages of 22–24 months [4]. Another study conducted with Ethiopian children also supports our findings, which determined the association of the odds on child underweight and severely underweight with age of children [60]. The reason for increased risk of underweight associated with age may be that as children’s age increased, they consumed complementary food along with breast milk. Contamination is more likely in complementary food, therefore implicated in spreading infectious diseases among children and in turn rendering them vulnerable to the risk of underweight [61].

Moreover, there is strong evidence that the prevalence of childhood disease is associated with child age, particularly in most of the developing countries [62]. Children living in higher wealth index households had smaller risk of being underweight and the finding was supported by other research work that claimed that children in lower wealth index households had greater chance of underweight than the children in higher wealth index households [56, 61, 63–66]. The explanation could be that children in higher wealth index households were more likely to belong to relatively food secure families, had comparatively more educated fathers and mothers, and lived in better neighborhood with improved health facilities. These factors might possibly have reduced the risk of underweight among the children living in higher wealth index households. It is widely known that maternal knowledge on child health has profound impact on child well nourishment, and a study performed in Bangladesh documented that mothers with better child health knowledge had mostly come from the richest households [67]. It implies the significance of household economic condition on child nutrition through improved maternal knowledge on child health. Respondents who reported their children had drunk plain water yesterday had lower odds on underweight for their children but there was no relationship between underweight and the type of toilet facilities. The risk of underweight was similar between male and female children, and between rural and urban children, which was unexpected. One explanation for this may be that poor people from rural areas were migrating to urban slum areas where economic conditions and health facilities were not remarkably different from rural areas. The risk for children to be underweight depended on the division they lived in. Children from the Sylhet division had higher probability of underweight relative to children from the Dhaka division. The reason is not clear why children living in the Sylhet division were at more risk of underweight. Nevertheless, one possible reason might be that poor socioeconomic conditions such as few skilled workers, limited exposure to media and poor maternal health were responsible for increased risk and higher prevalence of underweight among under five children in the Sylhet division [4].

The study had some limitations that should be addressed. First of all, the study was cross-sectional in design indicating that it was impossible to establish any causal relationship between underweight and the estimated risk factors. Nevertheless, the findings have great significance as it is claimed that the results from the same specific area using cross-sectional data have very limited variation over time. Secondly, household income is a well-established determinant of child underweight. But it is nearly impossible to have reliable data on household income and expenditure, particularly in developing countries such as Bangladesh. That being the case, we elected to use wealth index as a proxy indicator of household income, bearing in mind that wealth index might not sufficiently represent household income.

Finally, data did not include child birth weight, mother’s weight, father’s weight, parents’ age, father’s occupation, or mother’s occupation. The magnitude of estimated risk factors could possibly be changed if those factors were adjusted for our regression model. Despite the study’s limitations, its strength was in assessing a large number of samples, hence the findings can be generalized to all Bangladeshi children below 5 years old in 2011–2012. This group of children needs to be re-examined in 5 and 10 years to determine whether underweight and severe underweight leads to lasting detriments for these children.

## Conclusion

Overall, in Bangladesh approximately one-third of the study population is estimated as underweight. Underweight prevalence not only reflects poor nutritional conditions for children in a country, in general, it also reflects high socio-economic costs for low quality of life, vulnerability to different diseases and greater risk of childhood death. Due to the fact that it is more likely that underweight children who survive to adulthood will experience greater health problems, the prevalence of underweight may also contribute to loss of productivity.

The study revealed that the Sylhet division has the highest risk of severe underweight for under five children, followed by the Chittagong division. With both divisions their poverty rates are among the lowest in the country according to the latest Bangladesh Poverty Maps. This paper reveals that other than household position in the lower wealth index, lower parental education is one of the most important risk factors for child underweight. Sylhet is the home of most Bangladeshis that migrate to the United Kingdom and are engaged in unskilled or semi-skilled blue-collar jobs and send remittances. Although remittances have improved land and housing in Sylhet, investment in other social sectors like education or business is reported to be insignificant [68]. In terms of other important social indicators like ‘parental education’ Sylhet is reported to be poor-performing, according to the Bangladesh Bureau of Statistics, the overall literacy rate in the Sylhet division was the lowest in the country, at 39.2% in 2007, with an even lower female literacy rate of 35.1% [69]. This result calls for government and non-government interventions to increase education nationwide as much as possible, especially for the Sylhet division. This will also markedly increase the mother’s age when children are born, and decrease family size, which are particularly important for reducing the prevalence of underweight children.

## Footnotes

1 Stunting: height for age < − 2 standard deviations (SD) of the WHO Child Growth Standards median

2 Wasting: weight for height < − 2 standard deviations (SD) of the WHO Child Growth Standards median

3 Underweight: weight for age < − 2 standard deviations (SD) of the WHO Child Growth Standards median
